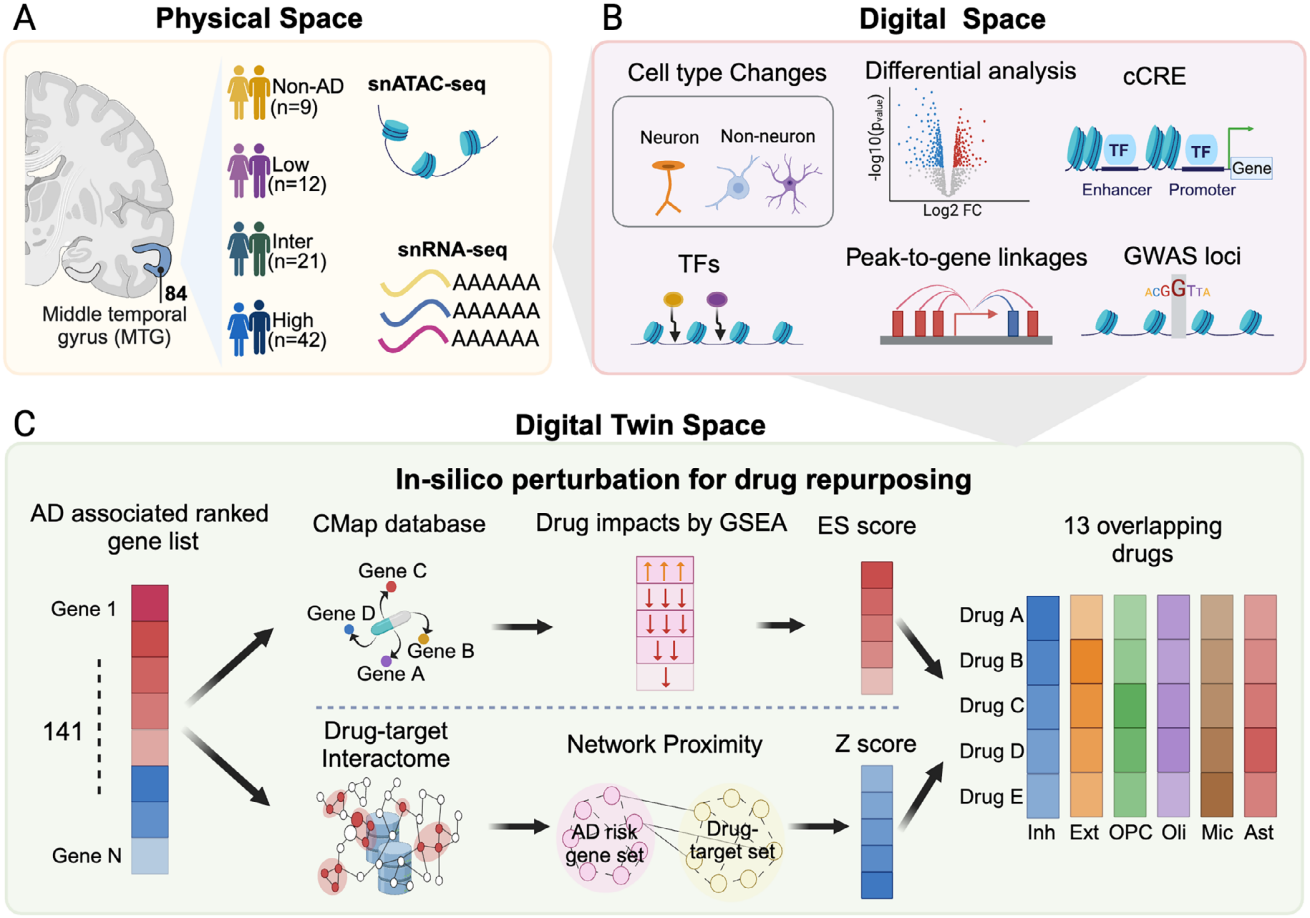# Single‐cell digital twins identify drug targets and repurposable medicine in Alzheimer’s disease

**DOI:** 10.1002/alz70859_102982

**Published:** 2025-12-25

**Authors:** Yunxiao Ren, Andrew A. Pieper, Jeffrey L. Cummings, Feixiong Cheng

**Affiliations:** ^1^ Cleveland Clinic Lerner Research Institute, Cleveland, OH USA; ^2^ Louis Stokes VA Medical Center, Cleveland, OH USA; ^3^ Case Western Reserve University, Cleveland, OH USA; ^4^ Louis Stokes Cleveland VA Medical Center, Cleveland, OH USA; ^5^ Brain Health Medicines Center, Harrington Discovery Institute, University Hospitals Cleveland Medical Center, Cleveland, OH USA; ^6^ Chambers‐Grundy Center for Transformative Neuroscience, Kirk Kerkorian School of Medicine, University of Nevada, Las Vegas, NV USA; ^7^ Cleveland Clinic, Cleveland, OH USA; ^8^ Cleveland Clinic Genome Center, Cleveland, OH USA

## Abstract

**Background:**

Alzheimer’s disease (AD) is a complex and poorly understood neurodegenerative disorder with a lack of effective treatments. Novel approaches for identifying FDA‐approved drugs with potential for AD treatment hold promise to address this challenge. One promising approach involves leveraging digital twins (DTs), which are virtual representations of physical entities by integrating advanced technologies such as artificial intelligence (AI), statistical modeling, network medicine, and multi‐omics analysis. DTs enable real‐time characterization of disease heterogeneities through continuous feedback and dynamic model updates, facilitating the identification of therapeutic targets.

**Method:**

In this study, we developed a single‐cell Digital Twin (scDT) framework to model AD progression by integrating single‐nuclei RNA‐seq (snRNA‐seq; 1,197,032 nuclei) and ATAC‐seq (snATAC‐seq; 740,875 nuclei) data from the middle temporal gyrus (MTG) of 84 donors across four stages (non‐AD, low, intermediate, and high) of AD neuropathological changes (ADNC) from the SEA‐AD cohort. This framework enabled prioritization of AD‐associated genes and identification of potential candidate drugs through a combination of gene set enrichment analysis (GSEA) and network proximity analysis.

**Result:**

We annotated six major cell types for snRNA‐seq and snATAC‐seq and observed distinct gene expression dynamics among these six major cell types during the early stages of ADNC, with these differences becoming more pronounced in high ADNC. By constructing cell type‐specific transcription factor (TF)‐target gene regulatory networks through leveraging peak‐to‐gene linkages and motif enrichment analyses, we identified cell type‐specific candidate *cis*‐regulatory DNA elements (cCREs). Integration of genome‐wide association study (GWAS) loci with cell type‐specific cCREs revealed 141 ADNC‐associated genes, including 36 genes targeted by FDA‐approved or clinically investigational therapies (e.g., ***ACE, ABCA1***, and ***CRHR1***). Furthermore, GSEA and network proximity analyses highlighted 13 repurposable drugs (e.g., galantamine, mecamylamine, dextromethorphan, tubocurarine, gabapentin, ebselen, resveratrol, imatinib, dipyridamole, deferoxamine, gemfibrozil, lansoprazole, and probucol) associated with these ADNC‐linked genes.

**Conclusion:**

In summary, we demonstrated a single‐cell digital twin framework for cell type‐specific target identification and drug repurposing in AD and other AD‐related dementia if broadly applied.